# Attenuation of synaptic toxicity and MARK4/PAR1-mediated Tau phosphorylation by methylene blue for Alzheimer’s disease treatment

**DOI:** 10.1038/srep34784

**Published:** 2016-10-06

**Authors:** Wenchao Sun, Seongsoo Lee, Xiaoran Huang, Song Liu, Mohammed Inayathullah, Kwang-Min Kim, Hongxiang Tang, J. Wesson Ashford, Jayakumar Rajadas

**Affiliations:** 1 Biomaterial and Advanced Drug Delivery Lab, Stanford University School of Medicine, Stanford, California, USA; 2 Gwangju Center, Korea Basic Science Institute, Gwangju 61186, Korea; 3 War Related Illness and Injury Study Center (WRIISC), VA Palo Alto Health Care System, Palo Alto, California, USA

## Abstract

Alzheimer’s disease (AD) is a neurodegenerative disease characterized by genotypic and phenotypic heterogeneity. Critical components of the two AD pathological pathways, Aβ-amyloidosis and Tauopathy, have been considered as therapeutic targets. Among them, much effort is focused on aberrant Tau phosphorylation and targeting Tau-phosphorylating kinases. Methylene blue (MB), a phenothiazine dye that crosses the blood-brain barrier, has been shown to hit multiple molecular targets involved in AD and have beneficial effects in clinical studies. Here we present evidence that microtubule affinity-regulating kinase (MARK4) is a novel target of MB. MB partially rescued the synaptic toxicity in *Drosophila* larva overexpressing PAR1 (MARK analog). In 293T culture, MB decreased MARK4-mediated Tau phosphorylation in a dose dependent manner. Further studies revealed a two-fold mechanism by MB including down-regulation of MARK4 protein level through ubiquitin-proteasome pathway and inhibition of MARK4 kinase activity *in vitro*. This study highlights the importance of MARK4 as a viable target for Tauopathy and provides fresh insight into the complex mechanism used by MB to treat AD.

Alzheimer’s disease (AD) is the most common neurodegenerative disease and causes progressive loss of memory and other cognitive functions. The pathological hallmarks of AD include amyloid plaque and neurofibrillary tangles (NFT). Tau, a highly soluble microtubule-binding protein, has been determined to be the major component of NFT, where it is hyperphosphorylated and becomes insoluble and filamentous^1^,^2^. Many kinases have been identified to regulate Tau phosphorylation, including microtubule affinity-regulating kinase (MARK), glycogen synthase kinase (GSK)-3β, and CDK5^3^,^4^. In *Drosophila*, overexpression of PAR1, the fly homolog of MARK kinase, leads to elevated tau phosphorylation at S262 and S356 sites and increased toxicity. More importantly, the initial phosphorylation of Tau by PAR1 at S262/S356 sites is prerequisite for the subsequent multisite phosphorylation of Tau by other kinases, such as GSK-3β and CDK5^5^. In addition, a genetic screen to identify modifiers of Tauopathy in a *Drosophila* model revealed that kinases and phosphatases are the major factors to affect Tau toxicity^6^. These studies suggest that orderly phosphorylation of Tau increases its toxicity and pharmacological intervention of these phosphorylation reactions may represent novel therapeutic strategy for AD treatment^7^.

Methylene blue (MB) is a dye that has been widely used in a range of different fields, including uses as a medicine in treating various bacterial and viral infections and cancer^8^. Recently it has been suggested that MB may have a beneficial effect on the cognitive performance of patients with AD^9^,^10^,^11^ possibly by inhibiting Tau protein aggregation, although this mechanism is still under debate^12^,^13^,^14^. Recent studies also suggested other possible mechanisms for the protective functions of MB in neurons, including reducing mitochondrial superoxide production^15^, enhancing mitochondrial function^16^, upregulating Nrf2/ARE genes^17^, modulating molecular chaperone activity^18^ and inducing autophagy^19^. However, the effect of MB on key Tau kinases has not been explored.

Here we studied the effect of MB on MARK4 mediated Tau phosphorylation in a *Drosophila* model as well as in mammalian cell cultures. A demethylated analog of MB, Azure C (AC) was also tested. In a screening study, both AC and MB showed activity in regulating Tau stability through heat shock protein Hsp70^18^. Olsalazine sodium (OS), an aminosalicylate drug used to treat inflammatory bowel disease was also included as a negative control. We found that MB may function through targeting the Tau kinase MARK4, resulting in reduced Tau phosphorylation at MARK4 sites. We also showed that this novel mechanism of MB involves both inhibition of MARK4 kinase activity and down regulation of MARK4 protein level.

## Results

### Methylene blue protects against PAR1 overexpression at neuromuscular junction (NMJ) in *Drosophila*

**Figure 1:**
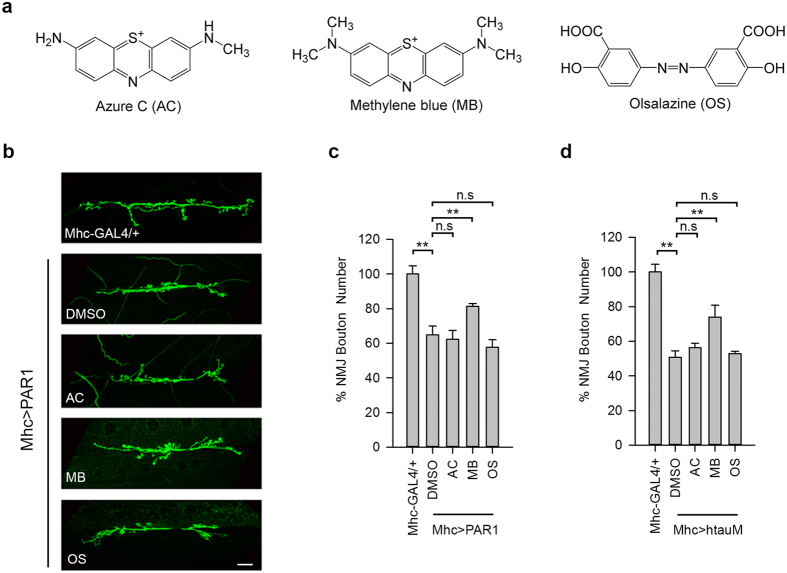
MB partially rescues bouton number loss at NMJ in PAR1 and htauM OE larvae. (**a**) Chemical structure of AC, MB and OS. (**b**) Immunostaining of wild-type (Mhc-GAL4/+) and PAR1 OE (Mhc > PAR1) third instar larval NMJs with anti-HRP antibodies. PAR1 OE larvae were fed with vehicle or drugs as indicated in the panel. Scale bar = 5 μm. (**c**,**d**) Statistical analysis of NMJ bouton number. 30 Wild-type and 25–35 Mhc > PAR1 (**c**) or Mhc > htauM (**d**) larvae treated with vehicle and drugs were analyzed. **0.001 < P < 0.01.

**Figure 2:**
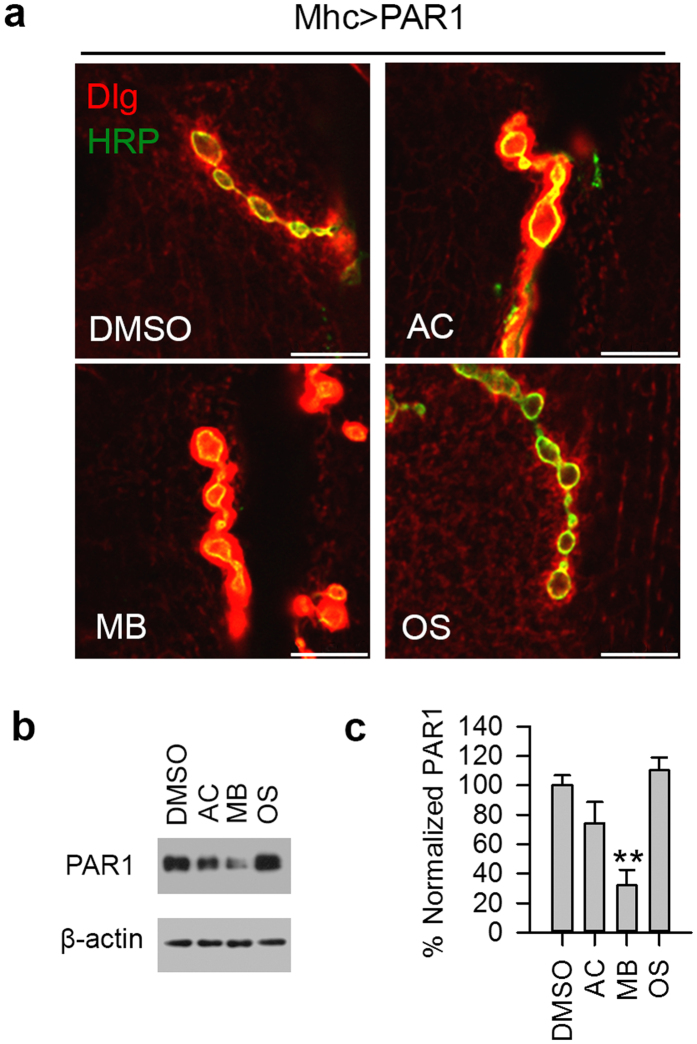
MB affects PAR1 function and protein level at NMJ in PAR1 OE larvae. (**a**) High-magnification views of the distribution patterns of Dlg at larval NMJ. NMJs of vehicle and drug treated PAR1 OE larvae were double-labeled with anti-Dlg (red) and anti-HRP (green) antibodies. Scale bar = 2 μm. PAR1 OE larvae were fed with vehicle or drugs as indicated. (**b**) MB reduces PAR1 protein level in larval muscle walls. Muscle walls were dissected out from late third instar larvae and homogenized for WB analysis. Representative immunoblot showing PAR1 protein level with β-actin as loading control. (**c**) Quantification of PAR1 level normalized to β-actin presented as percent of vehicle treated control (n = 3). **0.001 < P < 0.01.

To determine whether AC, MB and OS (Fig. 1a) can affect PAR1 function *in vivo*, we used *Drosophila* NMJ as the model system. Previously, it has been shown that PAR1/MARK is predominantly localized at NMJ and overexpression (OE) of PAR1 leads to decreased synapse formation and synaptic transmission^20^,^21^, which is mediated by Tau^22^. PAR1 OE also leads to the mislocalization of PSD-95/Dlg protein, a major synaptic substrate of PAR1^21^. To evaluate the effect of drug treatment on synapse formation, the number of type I boutons, an excitatory glutamatergic synapse, was determined by immunostaining of larval NMJ using the anti-horseradish peroxidase (HRP) antibody as described previously^21^,^23^. Interestingly, when we fed larvae with MB at 20 μM, MB effectively rescued bouton number loss in larvae overexpressing PAR1 or human tau R406W (htauM, a pathogenic form of tau related to tauopathy) under the control of Mhc-Gal4 driver (Fig. 1b–d). In comparison, AC and OS showed little effect (Fig. 1b–d). Dlg localization was examined by double staining of larval NMJ using the anti-HRP and anti-Dlg antibodies. In PAR1 OE larvae, Dlg was diffusive and less focused to the postsynapse (Fig. 2a) as reported previously^21^. Both AC and MB treatment rescued the mislocalization of Dlg at NMJ, while OS was not effective (Fig. 2a). To test whether MB directly affects PAR1 protein level, larval muscle walls were homogenized and PAR1 levels were analyzed by Western blot (WB). As shown in Fig. 2b,c, PAR1 protein level at NMJ decreased after MB treatment compared to vehicle treated controls, while AC and OS were less effective. This effect of MB treatment is specific because MB failed to alter the protein level of another pathogenic kinase human leucine-rich repeat serine/threonine-protein kinase 2 (hLRRK2) (Fig. S1). These results suggest that MB may affect PAR1 activity as well as protein level *in vivo*.

### Methylene blue reduces Tau phosphorylation in mammalian cell cultures

**Figure 3:**
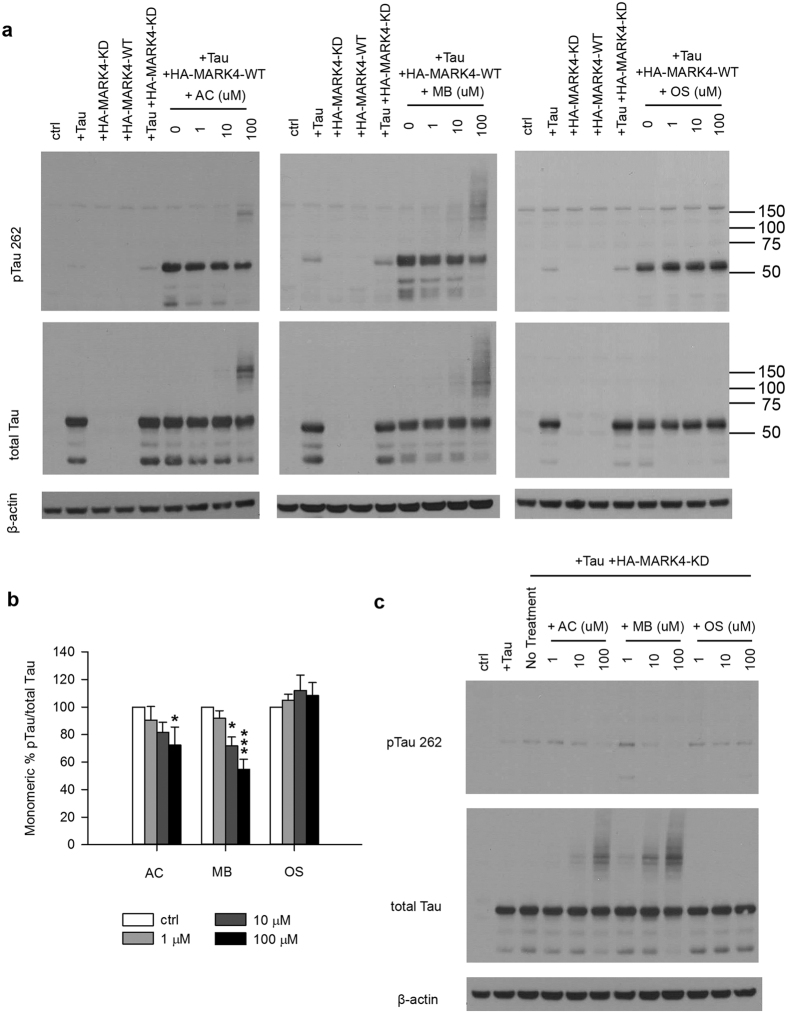
MB reduced Tau phosphorylation by overexpressed MARK4 in 293T cells. (**a**) Representative immunoblot of lysates from 293T cells transfected with 4R2N Tau alone, HA-MARK4 (KD and WT) alone or Tau + HA-MARK4. Cells transfected with Tau + HA-MARK4-WT were treated with AC, MB and OS for 3 h at concentrations indicated in the panel. The levels of pTau S262 and total Tau were reported with β-actin as the loading control. (**b**) Quantification of the ratio of monomeric pTau S262 to monomeric total Tau presented as the percent of the level found in cells transfected with Tau and MARK4-WT but without drug treatment. Results are mean ± SD (n = 3). *0.01 < P < 0.05; ***P < 0.001. Student’s t test is conducted between corresponding values in AC and OS or MB and OS group. (**c**) Representative immunoblot of lysates from 293T cells transfected with Tau + HA-MARK4-KD followed by treatment with AC, MB and OS as described in A). β-actin blots were cropped. Full blots are shown in Supplementary Fig. S4.

To extend our observation to mammalian system, we tested whether MB has inhibitory effect on MARK4 mediated Tau phosphorylation in mammalian cell cultures. 293T cells were transiently transfected with plasmids encoding human 4R2N Tau and HA tagged MARK4 (HA-MARK4-WT). A kinase dead form of MARK4 (HA-MARK4-KD) was included as a control. As shown in Fig. 3a, Tau expression was detected only after transfecting the cells with mammalian Tau construct. A low level of Tau phosphorylation at the MARK4 site serine 262 (pTau S262) was observed even without co-transfection of HA-MARK4-WT, indicating an activity from endogenous MARK4 (Fig. 3a). The level of pTau S262 was markedly increased when the cells were co-transfected with HA-MARK4-WT but not HA-MARK4-KD. After incubation of transfected cells with increasing concentrations of MB for 3 hours, pTau S262 level was reduced in a dose dependent manner as shown by WB analysis (Fig. 3a,b). No significant change of total Tau protein level was observed (Fig. 3a). AC also inhibited Tau phosphorylation at S262, but to a lesser extent while OS had no effect, even at 100 μM.

It is worth noting that we also detected high molecular weight forms of Tau (HMW Tau) when the cells were treated with high concentrations of MB and AC. The HMW Tau was seen as protein bands/smear larger than 75 kDa and appeared in both pTau and total Tau blots (Fig. 3a). One possibility is that phosphorylated Tau is more susceptible to MB treatment and selectively sequestered in HMW forms, resulting in apparent reduction in monomeric pTau. To test this possibility, we repeated the experiment with HA-MARK4-KD. If the HMW Tau preferentially consists of pTau but not unphosphorylated Tau, it will not be detected when the cells are co-transfected with HA-MARK4-KD. As shown in Fig. 3c, a low level of Tau S262 was phosphorylated by endogenous MARK4 but no phosphorylated HMW Tau was observed in the absence of HA-MARK4-WT overexpression. However, HMW Tau were still detected with increasing concentrations of MB or AC in the total Tau blot (Fig. 3c). Therefore, MB and AC seem to cause the formation of HMW Tau irrespective of the phosphorylation state of Tau. The physiological significance of the HMW Tau formation after MB and AC treatment is not clear. Nonetheless, densitometry analysis indicates that the ratio of monomeric pTau S262/total Tau was decreased with MB or AC treatment (Fig. 3b). HMW Tau amounts to 18–27% of the total signal. Since HMW Tau was observed in both pTau and total Tau blots, including HMW Tau in the quantification did not significantly change their ratio and the final results.

**Figure 4:**
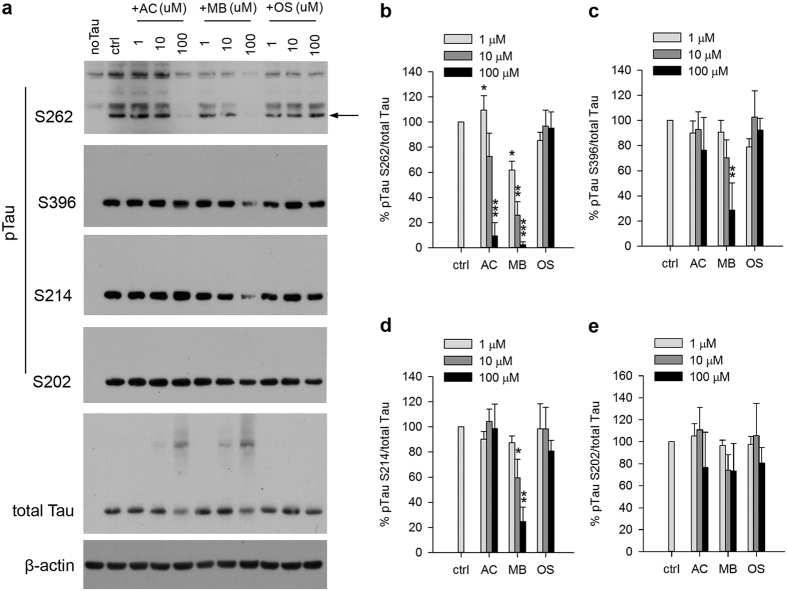
MB reduced Tau phosphorylation by endogenous kinases in 293T cells. (**a**) Representative immunoblot of lysates from 293T cells transfected with 4R2N Tau. Transfected cells were treated with AC, MB and OS for 3 h at concentrations indicated in the panel. The levels of pTau S262, S396, S214, S202 and total Tau were reported with β-actin as the loading control. (**b–e**) Quantification of the ratio of pTau to total Tau presented as the percent of the level found in cells transfected with Tau but without drug treatment. Results are mean ± SD (n = 3). *0.01 < P < 0.05; **0.001 < P < 0.01 ***P < 0.001. Student’s t test is conducted between corresponding values in AC and OS or MB and OS group.

Since endogenous MARK4 activity was detected, we repeated the experiment under more physiologically relevant condition without overexpression of HA-MARK4. Similar results were observed as shown in Fig. 4a,b where Tau phosphorylation at S262 by endogenous MARK4 was inhibited by MB in a dose dependent manner. AC had a similar effect, but to a lesser extent whereas OS had no effect (Fig. 4a,b). Longer exposure was used to visualize the weaker phosphorylation signal by endogenous kinase, resulting in some nonspecific bands (Fig. 4a).

To evaluate the selectivity of kinase inhibition by these compounds, we also measured Tau phosphorylation by other endogenous kinases at additional sites including Serine 202, 214 and 396. GSK-3β and CDK5 among other kinases have been shown to be involved in Tau phosphorylation at these sites^24^. Without any drug treatment, Tau was found to be phosphorylated at these sites. Interestingly, MB inhibited Tau phosphorylation at S396 and S214 but with reduced potency compared to the inhibition of pS262. No significant effect on pS202 was found by MB treatment (Fig. 4a,c–e). In contrast, AC and OS did not significantly affect the phosphorylation at any of these additional sites (Fig. 4).

### MB down-regulates MARK4 protein level in mammalian cell cultures

**Figure 5:**
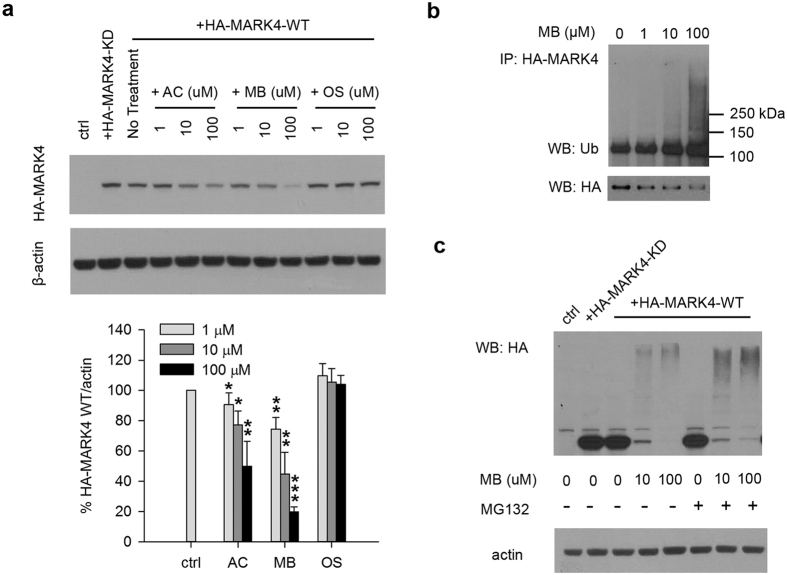
MB down regulates MARK4 protein level through ubiquitin-proteasome degradation in 293T cells. (**a**) Representative immunoblot of lysates from 293T cells transfected with HA-MARK4-KD or WT. Cells transfected with HA-MARK4-WT were treated with AC, MB and OS as described in Fig. 2a. MARK4 protein level was evaluated using an anti-HA antibody with β-actin as the loading control. Lower panel shows the quantification of HA-MARK4-WT normalized to β-actin presented as the percent of the level found in cells transfected with MARK4-WT but without drug treatment. Results are mean ± SD (n = 3). *0.01 < P < 0.05; **0.001 < P < 0.01; ***P < 0.001. Student’s t test is conducted between corresponding values in AC and OS or MB and OS group. (**b**) Representative immunoblot of immunoprecipitated HA-MARK4 probed by ubiquitin antibody (WB: Ub) and HA antibody (WB: HA). HA-MARK4 was immunoprecipitated from lysates of 293T cells transfected with HA-MARK4 followed by incubation with increasing concentrations of MB for 3 h. (**c**) Representative immunoblot of HA-MARK4 from lysates of 293T using an anti-HA antibody with β-actin as the loading control. Cells transfected with HA-MARK4 were treated with increasing concentrations of MB for 3 h in the presence of absence of 500 nM of proteasome inhibitor (R)-MG132. All blots were cropped. Full blots are shown in Supplementary Fig. S3.

To test whether MARK4 stability is affected by treatment of the drugs in mammalian cell cultures, 293T cells are transfected with HA-MARK4 and protein levels are examined by WB. Similar to what we observed in the *Drosophila* model, HA-MARK4-WT protein level was down regulated by MB in a dose dependent manner (Fig. 5a). AC had the same effect but less potent while OS did not affect HA-MARK4-WT protein level at all concentrations (Fig. 5a). To further confirm this effect of MB on MARK4 protein stability, the WB was repeated using a MARK4 antibody instead of an HA antibody. This MARK4 antibody appears to only react with endogenous MARK4 even in the presence of exogenous HA-MARK4. As shown in Supplementary Fig. S2a, MB also down-regulates endogenous MARK4 protein level in a dose dependent manner. Similarly, AC reduced endogenous MARK4 protein level, but to a lesser extent, while OS showed no effect (Fig. S2a). To determine whether this protein level down regulation by MB is a global effect or an effect specific to MARK4, we examined by WB the endogenous protein levels of GSK-3β and CDK5. We found no change of protein levels of either of these two kinases at all concentrations of MB as well as AC and OS (Fig. S2b), indicating the effect of MB in 293T cells is specific to MARK4.

Given the known function of PAR1 de-/ubiquitination in putative AD mechanisms^22^, we examined whether MB treatment leads to an increase of MARK4 ubiquitination. After MB treatment, HA-MARK4-WT was immunoprecipiated and its ubiquitination was analyzed by WB using an anti-Ub antibody. As shown in Fig. 5b, an increase of high molecular weight smear was caused by MB treatment in a dose dependent manner, indicating an increase of HA-MARK4-WT ubiqitination by MB. To further confirm ubquitinated HA-MARK4 is degraded by proteasome, the cells were pretreated with a proteasome inhibitor MG132 followed by MB treatment. When HA-MARK4-WT protein level was analyzed by WB, an accumulation of high molecular weight poly-ubiquitinated HA-MARK4-WT was observed in the presence of MG132, indicating that 26S proteasome complex is at least in part responsible for the increased MARK4 degradation (Fig. 5c).

### Methylene Blue inhibits MARK4 mediated Tau phosphorylation *in vitro*

**Figure 6:**
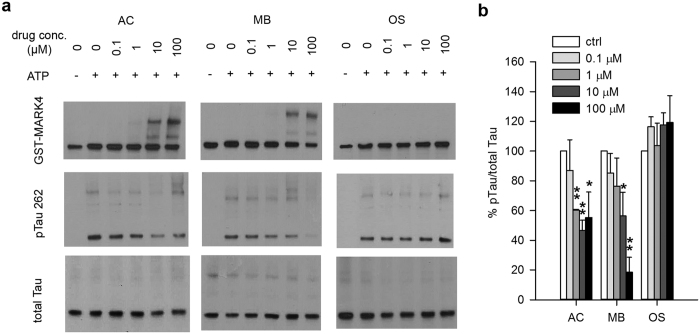
MB directly inhibits the ability of MARK4 to phosphorylate Tau in an *in vitro* kinase assay. GST-MARK4 was incubated with purified His-Tau and ATP in the presence of increasing concentration of AC, MB or OS for 30 min at 30 °C. (**a**) Representative immunoblot of the kinase reaction probed by antibodies against GST, pTau S262 and total Tau. (**b**) Quantification of the ratio of monomeric pTau S262 to monomeric total Tau presented as the percent of the level without drug treatment. Results are mean ± SD (n = 4). *0.01 < P < 0.05; **0.001 < P < 0.01. Student’s t test is conducted between corresponding values in AC and OS or MB and OS group.

To determine whether MB directly affects MARK4 kinase activity, we tested the phosphorylation of Tau by MARK4 in the presence or absence of MB using a cell-free kinase assay system. Purified recombinant GST-MARK4 from a commercial source was used. When MB was added directly to the kinase reaction, we observed a concentration dependent decrease of GST-MARK4 mediated Tau phosphorylation (Fig. 6a,b). Interestingly, this phosphorylation appeared to be inversely correlated with the formation of a higher molecular weight band which appears to be GST-MARK4 dimers (Fig. 6a). Inhibition of Tau phosphorylation and GST-MARK4 dimer formation was also observed with AC treatment (Fig. 6a,b). When OS was tested under the same condition, no inhibition of GST-MARK4 mediated Tau phosphorylation was observed (Fig. 6a,b). Neither was any GST-MARK4 dimer formation.

To confirm the results of this kinase assay and investigate whether this dimer formation is dependent on the GST tag, the kinase assay was repeated with MARK4 obtained by immunoprecipitation from 293T cells overexpressing the HA tagged kinase instead of GST-MARK4. As shown in Supplementary Fig. S3, immunoprecipitated HA-MARK4-WT effectively phosphorylated Tau at S262 site as shown by WB, while HA-MARK4-KD failed to do so. The same dose dependent inhibitory effect of MB on Tau phosphorylation was observed (Supplementary Fig. S3). Increased formation of HA-MARK4-WT dimer with increased concentration of MB was also observed, indicating that the dimer formation is independent of the tag.

Some HMW Tau was also observed (Fig. 6a). However, it was detected even without drug treatment and the slight change of its density did not correlate with the concentration of the drugs. Therefore, the decreased Tau phosphorylation at S262 upon treatment with MB and AC cannot be accounted for by the HMW Tau formation.

## Discussion

It has been proposed that aberrant hyperphosphorylation of Tau -likely caused by the imbalance between kinase and phosphatase activities- is the initiating event of a pathogenic cascade that leads to neuronal death^7^. More than two dozen protein kinases phosphorylate Tau *in vitro* and *in vivo*^25^,^26^. Therefore a plausible and desirable therapeutic target would be to inhibit Tau kinases. Among those kinases, CDK5, GSK-3β and MARK have been proposed as the three major kinases responsible for phosphorylating the majority of epitopes that are present in paired helical filament (PHF) form of Tau^7^,^25^,^27^. Selective and non-selective inhibitors of CDK5 and GSK-3β have been developed^26^,^28^. But none has shown beneficial effect for AD in clinical studies. Although MARK has been shown to play an important role in Tauopathy, no MARK specific inhibitor has been developed except for one study reporting the use of a peptide inhibitor derived from the CagA protein of Helicobacter pylori^20^. In this study, we provide evidence both *in vivo* and *in vitro* that MARK4 and its *Drosophila* homolog PAR1 are putative targets of MB. Previous studies have identified multiple targets of MB including Tau itself, but none has explored its effects on Tau kinases. Our mechanistic study revealed both down regulation of kinase protein level and inhibition of kinase activity. These findings emphasized the importance of Tau kinase MARK4 as a therapeutic target and identified novel mechanisms of action by MB as an AD drug.

Whether MB’s effect on Tau phosphorylation is specific to MARK4 site remains unclear. In transfected 293T cells, Western blot revealed that Tau phosphorylation by other kinases was also reduced by MB (Fig. 4). Nonetheless it showed that the inhibition of pS262 is the most potent compared to other kinase sites (Fig. 4). Since there is evidence that Tau phosphorylation at S262 is required for subsequent multisite phosphorylation^5^, it is possible that the reduced phosphorylation at these other sites is simply caused by the lack of the priming event, rather than direct inhibition by MB. To fully address this issue, a pseudophosphorylated Tau with a Serine 262 to Aspartate mutation can be used in future studies.

Another concern raised from the studies of transfected 293T cells is the observation of HWM Tau when cells were treated with high concentrations of MB. There is evidence that Tau oligomers are the toxic form of Tau in neurodegenerative disease^29^. Further study showed that Tau oligomerization in AD and related Tauopathies is hyperphosphorylation-dependent^30^. However, the formation of HWM Tau under the treatment of MB in this study is not hyperphosphorylation-dependent. As shown in Fig. 3c, unphosphorylated Tau also forms HWM bands when treated with MB. It will be interesting to examine the nature of the HWM Tau and its physiological role in future studies.

We provide evidence that down-regulation of MARK4 protein level by MB is specific and at least in part through degradation by the ubiquitin-proteasome system (Fig. 5). Lee *et al*. showed that phosphorylated PAR1 is targeted for ubiquitination and degradation by Slimb, the *Drosophila* SCF (Skp_Cul1_F-box) E3 ubiquitin ligase complex^22^. They also showed that Slimb’s action is antagonized by the deubiquitinase fat facets (FAF). In addition, Ub-specific protease 9X (USP9X), the putative mammalian homologue of FAF, was also identified to catalyze the removal of polyubiquitin chains from MARK4^31^. However, in this study, Lys(29)/Lys(33)-linked polyubiquitin chains seem to only affect MARK4 kinase activity, not stability. Based on these studies, a deubiquitinase is a possible target of MB through which the stability of MARK4 is regulated. Future studies may focus on whether MB affects USP9X or other deubiquitinases and which ubiquitination sites are involved. Other studies have shown that MB induces autophagy/macroautophagy resulting in direct reduction of Tau protein level or AMPK activation^19^,^32^. Interestingly, a recent study of hippocampal tissues from AD cases revealed colocalization of MARK4 and pTau S262 in granulovacuolar degeneration bodies (GVDs)-vacuoles with characteristics of autophagic system and correlation with AD brains^33^. Therefore, it is possible that increased autophagy induced by MB also facilitates the clearance of MARK4.

Our *in vitro* kinase assay results indicate that MB directly inhibits Tau phosphorylation by MARK4 at Ser262 (Fig. 6). An interesting observation is that inhibition seems to correlate with MARK4 dimerization which is also seen with AC but not OS. Regulation by dimerization is not a rare feature of kinases. In fact, there is evidence that Tau phosphorylation by MARK2 is suppressed by the heterodimerization of MARK2 with another kinase, PAK5^34^. Crystal structures have been determined for MARK1, 2 and 3, all consisting of homodimers^35^. The predicated structure of MARK4 has a similar domain organization^35^,^36^. Therefore, it is possible that homodimerization is a mechanism for MARK4 inhibition, though *in vivo* evidence is still lacking. Based on these reports, we speculate that MB may inhibit MARK4 by stabilizing its dimer, possibly by covalent cross-linking of dimerized MARK4. Previous studies focused on how MB affects Tau fibril formation have found that MB causes cysteine oxidization, resulting in modified cysteine residues and/or intra and intermolecular disulfide bonds^13^,^14^. Since the WB analysis in this study was performed under reducing condition, disulfide bonds formation is unlikely. The nature of this dimerization and its physiological relevance remains to be investigated.

Although PAR1-Tau axis has been shown to play a crucial role in AD processes, which Aβ toxicity and/or other triggers are likely to impinge on^20^, it seems that kinase inhibition alone or inhibition of one kinase is not sufficient to achieve better clinical outcome. A growing body of evidence suggests that MB has multiple molecular targets throughout AD pathways^9^,^13^,^14^,^16^,^17^,^18^,^19^,^32^. The identification of the novel target and mechanisms of MB reported in this study provides a more comprehensive understanding of its neuroprotective effect and contributes to the development of more efficient therapeutics/combination therapy for the multi-factorial Alzheimer’s disease.

## Materials and Methods

### Materials

Azure C was purchased from Fluka/Sigma (St. Louis, MO), methylene blue was purchased from EMD Chemicals (Gibbstown, NJ) and olsalazine sodium was purchased from AK Scientific (Union City, CA). Expression plasmid for the human full-length four-repeat Tau in *E. coli* expression vector pT7C was a kind gift of Dr. Lester I. Binder (Northwestern University). Mammalian expression constructs of Tau, HA-MARK4 WT and KD are generous gifts of Dr. Bingwei Lu (Stanford University). (R)-MG132 was purchased from Cayman Chemical (Ann Arbor, MI)

### *Drosophila* strains

The UAS-PAR1-Myc was described before^5^. Mhc-Gal4 driver was provided by Dr. J. Troy Littleton (Massachusetts Institute of Technology). UAS–hLRRK2–WT–Flag were obtained from Dr. W. Smith (Johns Hopkins University)^37^,^38^. For pharmacological approach, AC, MB or OS at 20 μM final concentration or DMSO was added to fly food. Feeding third instar larvae were collected from the drug-containing food and dissected in PBS. All flies were raised at 25 °C.

### Immunofluorescence imaging

Third instar larvae were selected, dissected in PBS, fixed in 4% formaldehyde (Ted Pella) in PBS for about 15 minutes and washed ×3 in 0.1% Triton X-100 in PBS. FITC-conjugated anti-HRP (Jackson ImmunoResearch Laboratories) was used at 1:150. For Dlg staining, monoclonal mouse anti-Dlg (1:500)^21^ and Alexa Fluor 594 nm conjugated goat anti-mouse IgG antibodies (Molecular Probes/Life Technologies) were used. Laval preparations were mounted in SlowFade Antifade kit (Invitrogen). Confocal images were collected from Leica TCS SP5 AOBS confocal microscopes equipped with 40× or 100× inverted NX oil lens, located at Korea Basic Science Institute (Gwangju, Korea). Leica Application Suite Advanced Fluorescence software was used to capture, process and analyze images. Analysis of the NMJ was performed essentially as described^21^.

### Cell culture

293T cells were cultured at 37 °C in 5% CO_2_ with Dulbecco’s modification of Eagle’s medium (DMEM) (Gibco/Life Technologies) with 10% fetal bovine serum (FBS) (Gibco/Life Technologies), 100 units/ml penicillin and 100 μg/ml streptomycin (Gibco/Life Technologies). For transfection, cells were plated in poly-D-lysine coated 12-well plates (BD) at 2.2 × 10^5^ cells/well. After 40 h incubation, each well were transfected with 1 μg of plasmid DNA encoding human 4R2N Tau and/or HA tagged human MARK4 (WT or KD) using lipofectamine 2000 (Life Technologies) according to manufacturer’s instruction. 24 h after transfection, cells were treated with AC, MB or OS at indicated final concentrations for 3 h at 37 °C. When MG132 was used to inhibit proteasomes, cells were pretreated with 500 nM MG132 for 1 h at 37 °C followed by drug treatment in the presence of MG132.

### Western blot

For fly larvae, dissected body-wall muscles were homogenized in lysis buffer (50 mM Tris pH 8.0, 1% Triton X-100, 150 mM NaCl, 2 mM Na_3_VO_4_, 10 mM NaF, 60 mM β-glycerolphosphate, 10% glycerol, protease inhibitor cocktail), and then centrifuged at 13,000 g for 20 minutes at 4 °C. Supernatants were boiled in SDS sample buffer. For 293T cultures, cells were washed in ice cold 1 × PBS and lysed in CHAPS lysis buffer (CLB: 50 mM HEPES pH 7.5, 150 mM NaCl, 1 mM EDTA, 0.3% CHAPS). After two free-thaw cycles, cell lysates were centrifuged at 13,000 rpm 4 °C for 10 min. Supernatant was used for BCA protein assay, immunoprecipitation or WB. Western blot was performed using the following primary antibodies: rabbit anti-PAR1 antibody^21^,^39^ at 1:3000; polyclonal rabbit anti-Tau (pS262) antibody (44750G, Life Technologies) at 1:2500; monoclonal mouse anti-Tau (pS396) (PHF13) (#9632, Cell Signaling) at 1:4000; monoclonal rabbit anti-Tau (pS214) (ab170892, Abcam) at 1:4000; monoclonal rabbit anti-Tau (pS202) (ab108387, Abcam) at 1:4000; polyclonal rabbit anti-human total Tau antibody (A0024, Dako) at 1:5000; monoclonal mouse anti-total GSK-3β (3D10) (#9832, Cell Signaling) at 1:2000; polyclonal rabbit anti-total CDK5 (#2506, Cell Signaling) at 1:2500; monoclonal mouse anti-HA antibody (H3663, Sigma) at 1:2500; monoclonal mouse anti-β-actin antibody (A1978, Sigma) at 1:10000; polyclonal rabbit anti-Glutathione-S-Transferase (GST) antibody (G7781, Sigma) at 1:2500; monoclonal mouse anti-ubiquitin (Ub) Antibody (P4D1) (sc-8017, Santa Cruz Biotech) at 1:500; and polyclonal rabbit anti-MARK4 antibody (bs-4659R, Bioss) at 1:3000. All primary antibodies were diluted in 5% BSA in 1 × TBST and incubated with the membrane at 4 °C overnight. Secondary antibodies goat anti-mouse IgG HRP conjugate (G21040, Life Technologies) and goat anti-rabbit IgG HRP conjugate (G21234, Life Technologies) were diluted in 1 × TBST and incubated with the membrane at room temperature for 1 h.

### Purification of recombinant 4R2N Tau

Purification of recombinant 4R2N hTau was carried out as described previously^41^,^41^. Briefly, a pT7C bacterial expression plasmid carrying the 4R2N hTau with an N-terminal His tag was expressed in *E. coli* strain BL21 (DE3). Recombinant protein was purified using Ni-NTA agarose (Qiagen).

### Kinase assay

For a typical reaction, 5 μl of 3 × kinase buffer (90 mM HEPES, 150 mM potassium acetate, 15 mM MgCl_2_) was mixed with 150 ng of GST-MARK4 (Sigma) and 150 ng of lab-made recombinant 4R2N hTau followed by addition of AC, MB or OS at the indicated concentrations. ATP was added last to a final concentration of 200 μM. The total volume was brought up to 15 μl with protease free H_2_O. The reaction was incubated at 30 °C for 30 min and then terminated by adding 4 × LDS buffer with 2-mercaptoethanol. After incubation at 70 °C for 10 min, the samples were analyzed by Western blot.

### Statistical analysis

Student’s t test (two tailed) was performed for statistical analysis.

## Additional Information

**How to cite this article**: Sun, W. *et al*. Attenuation of synaptic toxicity and MARK4/PAR1-mediated Tau phosphorylation by methylene blue for Alzheimer's disease treatment. *Sci. Rep.* **6**, 34784; doi: 10.1038/srep34784 (2016).

## Supplementary Material

Supplementary Information
