# Supplementary material for: Attenuation of synaptic toxicity and MARK4/PAR1-mediated Tau phosphorylation by methylene blue for Alzheimer’s disease treatment
Source: Sci Rep. 2016 Oct 6;6:34784. doi: 10.1038/srep34784 (PMC5052533; doi:10.1038/srep34784)
Supplement: Supplementary Information [file srep34784-s1.pdf]

Supplementary Methods and Figures

Attenuation of synaptic toxicity and MARK4/PAR1-mediated Tau phosphorylation by methylene blue for Alzheimer's disease treatment

Wenchao Sun[1†], Seongsoo Lee[1,2†], Xiaoran Huang[1†], Song Liu[1], Mohammed Inayathullah[1], Kwang-Min Kim[1], Hongxiang Tang[1], J. Wesson Ashford[3] and Jayakumar Rajadas[1*]

[1]Biomaterial and Advanced Drug Delivery Lab, Stanford University School of Medicine, Stanford, California.

[2]Gwangju Center, Korea Basic Science Institute, Gwangju 61186, Korea

[3]War Related Illness and Injury Study Center (WRIISC), VA Palo Alto Health Care System, Palo Alto, California

[†]These authors contributed equally to this work.

[*]Correspondence and requests for materials should be addressed to

Jayakumar Rajadas, Ph.D.

Supplementary Methods

Immunoprecipitation and kinase assay

Immunoprecipitation and kinase assay were performed as described previously[1] with minor modification. Briefly, 293T cells transfected with plasmids that carry wild type (WT) or kinase dead (KD) form of HA-MARK4 were lysed in ice-cold CHAPS lysis buffer (CLB) (50 mM HEPES pH 7.5, 150 mM NaCl, 1 mM EDTA, 0.3% CHAPS, 1 mM DTT, 1×Halt Protease and Phosphatase Inhibitor Cocktail (Thermo Scientific)). After two freeze-thaw cycle, the soluble fractions of the lysates were isolated by centrifugation at 13,000 rpm 4°C for 10 min. The concentration of total protein was determined using BCA Protein Assay Reagent (Thermo Scientific). For immunoprecipitation assay, all incubation was carried out at 4°C with rotation. In a typical assay, lysate which contains 1 mg of protein in 500 µl of CLB was pre-cleared by incubation with 12 µl of 50% slurry of protein G agarose (Thermo Scientific) for 1 h. In a separate microcentrifuge tube, 4 µl monoclonal mouse anti-HA antibody (Sigma) was bound to 12 µl of 50% slurry of protein G agarose in a total of 100 µl CLB for 1 h followed by the addition of the pre-cleared lysate and incubation overnight. Immunoprecipitates were washed three times with CLB, once with wash buffer (40 mM HEPES, 250 mM NaCl), and once with kinase buffer (30 mM HEPES, 50 mM potassium acetate, 5 mM $MgCl_2$). Agarose beads were resuspended in 200 µl kinase buffer with 200 µM of ATP. For each kinase reaction, 25 µl of resuspended agarose beads were taken, to which MB was added followed by recombinant Tau 441 (250 ng per reaction). The reaction was carried out at 30°C for 30 min, and terminated with 4×LDS buffer with 2-mercaptoethanol. After incubation at 70°C for 10 min, the samples were analyzed by Western blot.

Supplementary Figures

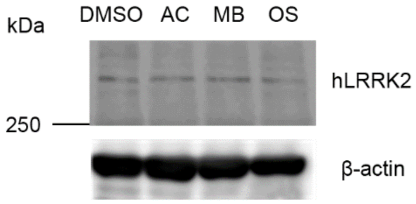

Supplementary Figure S1. MB does not affect human Leucine-rich repeat serine/threonine-protein kinase 2 (hLRRK2) protein level. Representative immunoblot showing hLRRK2 protein level with β-actin as loading control.

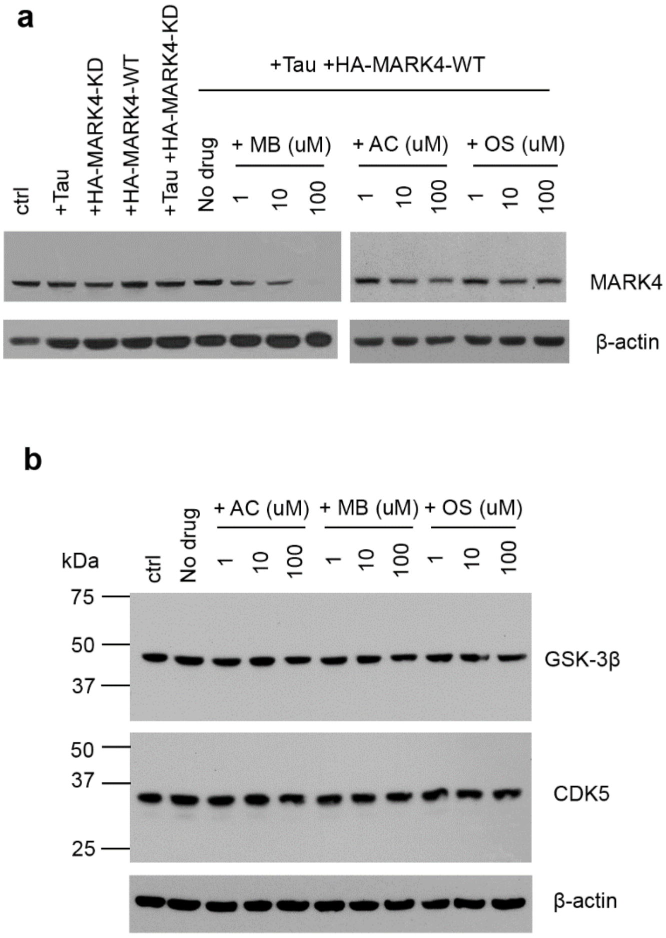

Supplementary Figure S2. MB down regulates endogenous MARK4 protein level but not GSK-3β and CDK5 in 293T cells. (a) Immunoblot of lysates from 293T cells transfected with 4R2N Tau and HA-MARK4-KD or WT. Cells transfected with Tau and HA-MARK4-WT were treated with AC, MB and OS as described in Figure 3A. Endogenous MARK4 protein level was evaluated using an MARK4 antibody with β-actin as the loading control. (b) Immunoblot of lysates from 293T cells transfected with 4R2N Tau. Transfected cells were treated with AC, MB and OS as described in Figure 4. All experiment was repeated twice and similar results were obtained. Full blots are shown in Supplementary Figure S5.

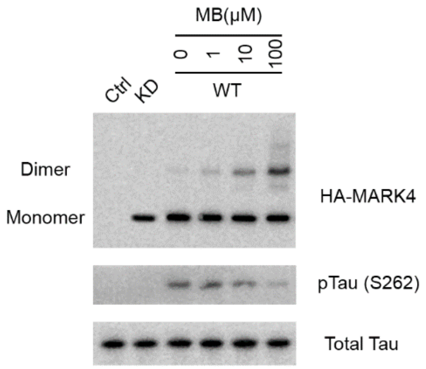

Supplementary Figure S3. ***In vitro*** **kinase assay using immunoprecipitated HA-MARK4.** 293T cells were transfected with HA-MARK4 WT and KD. HA-MARK4 was immunoprecipitated using anti-HA antibody and incubated with purified hTau and ATP in the presence of increasing concentration of MB. Representative immunoblot of the kinase reaction probed by antibodies against HA, pTau S262 and total Tau was shown. The experiment was repeated three times and similar results were obtained. Total Tau and pTau (262) blots were cropped. Full blots are shown in Supplementary Figure S5.

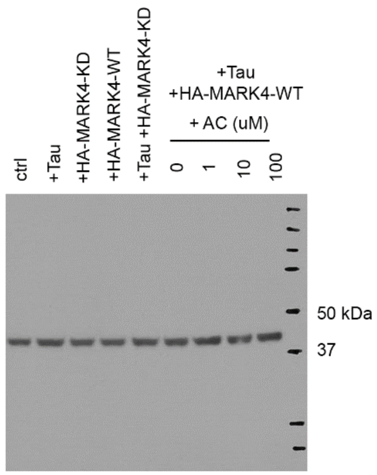

Fig 3a AC β-actin

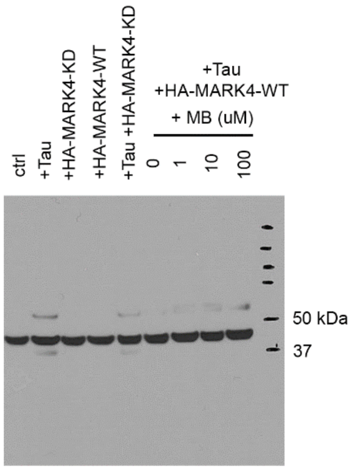

Fig 3a MB β-actin

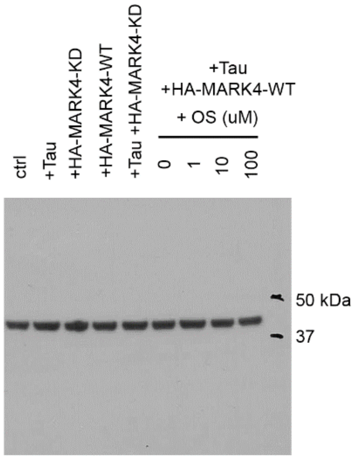

Fig 3a OS β-actin

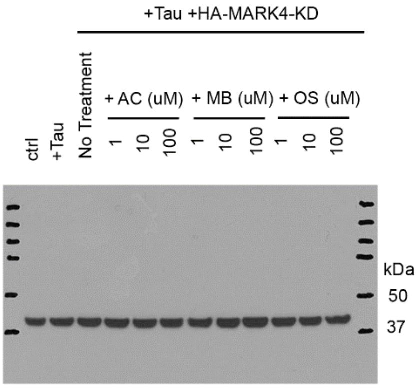

Fig 3c β-actin

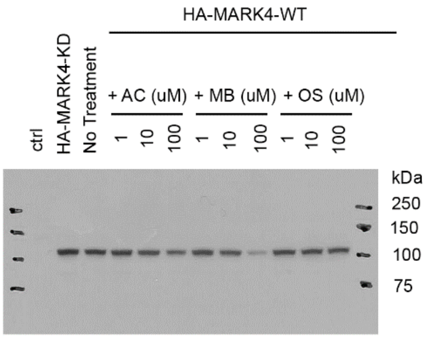

Fig 5a HA-MARK4

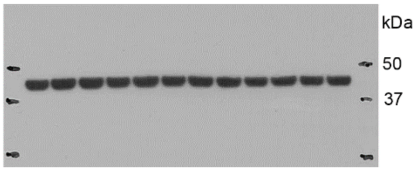

Fig 5a β-actin

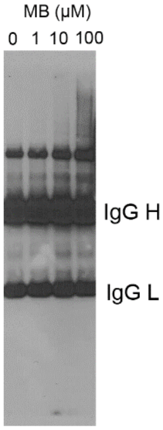

Fig 5b Ub

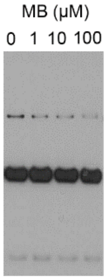

Fig 5b HA

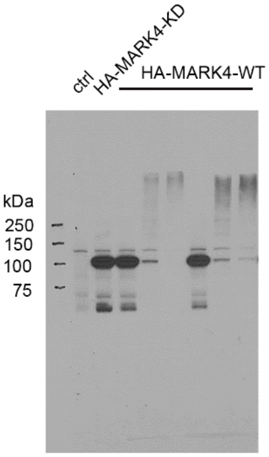

Fig 5c HA

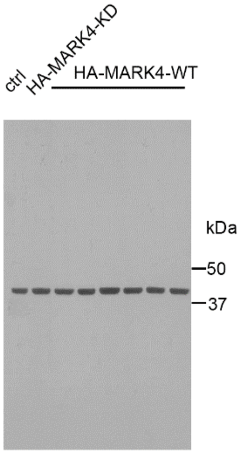

Fig 5c β-actin

Supplementary Figure S4. Full-length blots 1. Each blot is identified at the bottom. Additional bands in Fig 5b represent mouse IgG heavy chain (H) and light chain (L) used for immunoprecipitation.

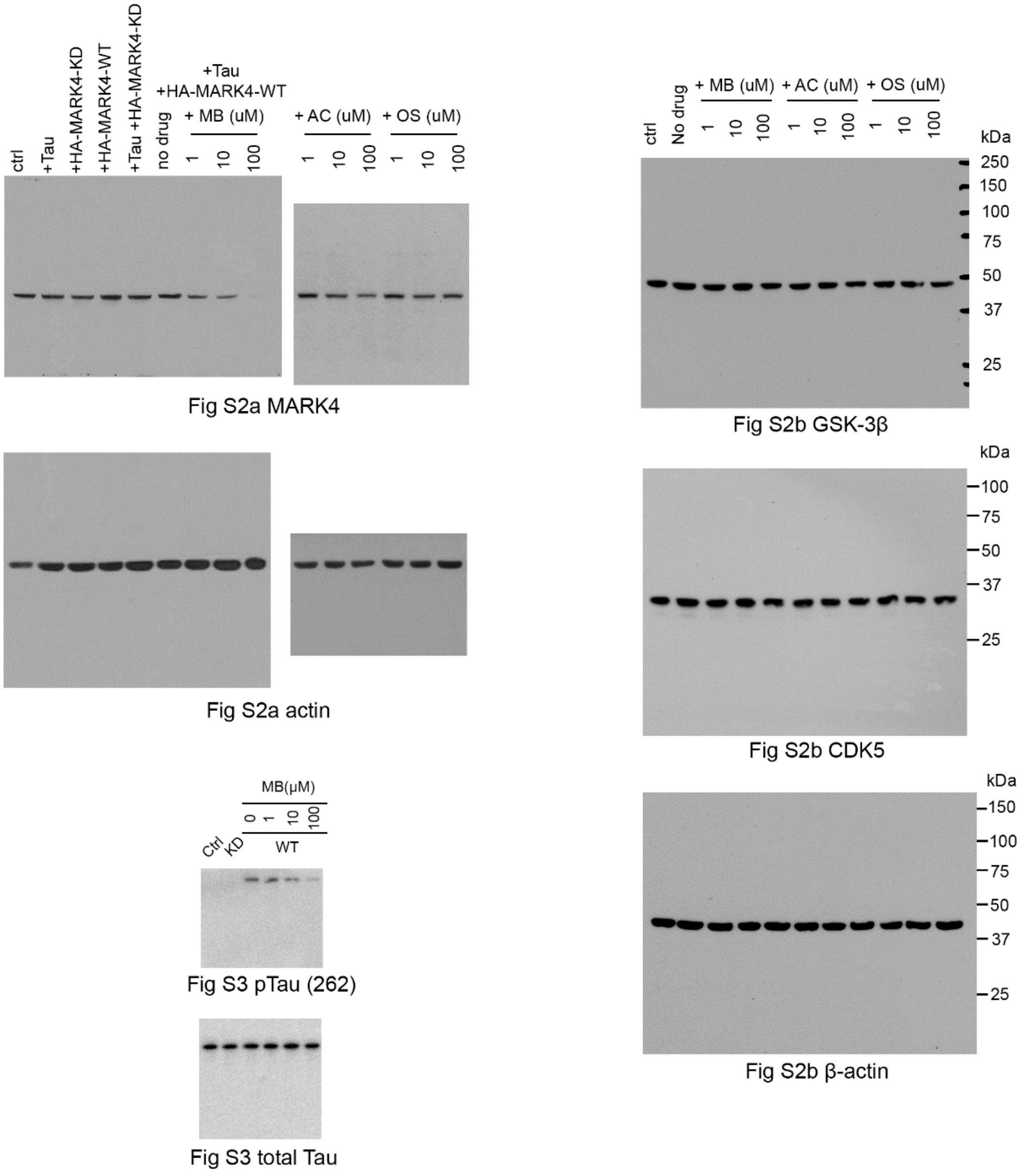

Supplementary Figure S5. Full-length blots 2. Each blot is identified at the bottom.

1. Li, L. & Guan, K. L. Microtubule-associated protein/microtubule affinity-regulating kinase 4 (MARK4) is a negative regulator of the mammalian target of rapamycin complex 1 (mTORC1). *J. Biol. Chem.* **288**, 703-708 (2013).
